# Reduced Serum Brain-Derived Neurotrophic Factor in Infants Affected by Severe Bronchiolitis

**DOI:** 10.2174/1570159X22999240223153901

**Published:** 2024-03-04

**Authors:** Raffaella Nenna, Carla Petrella, Enea Bonci, Paola Papoff, Margherita di Jorgi, Laura Petrarca, Maria Giulia Conti, Christian Barbato, Alessandra Pietrangeli, Marco Fiore, Fabio Midulla, BROME Group

**Affiliations:** 1 Department of Experimental Medicine, Medical Faculty, Sapienza University of Rome, Italy;; 2 Department of Sense Organs, Medical Faculty, Institute of Biochemistry and Cell Biology (IBBC-CNR), Sapienza University of Rome, Viale del Policlinico 155-00161, Rome, Italy;; 3 Virology Laboratory, Department of Molecular Medicine, “Sapienza” University, Rome, Italy;; 4 Department of Maternal Infantile and Urological Sciences, Sapienza University of Rome, Viale Regina Elena 324-00161, Roma, Italy

**Keywords:** Neurotrophin, BDNF, TrkB, Nfl, bronchiolitis, RSV, O_2_ supplementation

## Abstract

**Background:**

Bronchiolitis is an acute viral infection of the lower respiratory tract, typical of infants in their first year of life and causing hypoxia in the most serious cases. Bronchiolitis recognizes various demographic risk factors that are associated with greater clinical severity; however, no laboratory factors are yet able to correlate with the clinical severity. Neurotrophins as Brain-Derived Neurotrophic Factor (BDNF) are mediators of neuronal plasticity. BDNF is constitutively expressed in smooth muscle cells and epithelium of the lower respiratory tract, and as it is released during inflammatory conditions, serum levels may have a relevant role in the prognosis of infants with bronchiolitis.

**Objective:**

In the present pilot study, we aimed to disclose the presence of serum BDNF in infants hospitalized with bronchiolitis at discharge as a disease severity indicator.

**Methods and Results:**

Serum BDNF, measured at hospital discharge, was significantly lower in severe bronchiolitis (expressed as O_2_-supplemented infants). Furthermore, no changes were disclosed for the Tropomyosin receptor kinase B, the main BDNF receptor and neurofilament light chain, a biomarker of neuronal degeneration.

**Conclusion:**

Low serum BDNF in infants with severe bronchiolitis could be associated with a higher utilization by lung cells or with an altered production by lung cells. Therefore, further research is required to study if a decreased production or increased consumption of this biomarker is at the base of the above-mentioned findings.

## 1 INTRODUCTION

Bronchiolitis is an acute viral infection of the lower respiratory tract, typical of infants in their first year of life and representing the first cause of hospitalization in this age group [1, 2]. Respiratory viruses, particularly Respiratory Syncytial Virus (RSV), cause bronchiolitis and have a seasonal pattern, commonly occurring in the winter months,between November and March [1-3]. About 70% of all infants were defined to experience, every year, a viral respiratory tract infection in their first year of life. Of these, about 20% would develop symptomatic disease, and 2% would require intubation and mechanical ventilation [4]. Neurological morbidity may occur in previously healthy children admitted to a pediatric intensive care unit with bronchiolitis requiring mechanical ventilation [5-7]. Indeed, increasing findings support the view that RSV could attack the central nervous system, infecting brain cells, such as neurons, astrocytes, and microglia, promoting neuroinflammation. It has also been shown that RSV infection can elicit neurological manifestations, including behavioral impairment and cognitive alterations [8].

Risk factors for bronchiolitis include male sex, premature birth, absence of breastfeeding, the presence of underlying lung diseases and hemodynamically significant congenital heart diseases, and severe immunodeficiencies [9, 10]. The only protective factor known is the presence of breastfeeding. However, most children are usually healthy when they become infected [11, 12].

Bronchiolitis has been proven to be associated with wheezing recurrence in the 2 decades following the infection; further, it’s been estimated that 50% of children who have bronchiolitis will develop asthma later in life, with a different pathophysiology and prognosis as compared to asthmatic patients who did not experience a lower respiratory tract infection in infancy [13-15]. RSV infection tends to cause hyperactivity of the lower respiratory tract in affected infants, a mechanism that was demonstrated to be mediated by several molecules, among which neurotrophins, several cytokines, and leukotrienes [16-19].

Neurotrophins are a family of proteins involved in the differentiation and survival of neurons, and four highly conserved members of this family are well known for having a similar structure and function: brain-derived neurotrophic factor (BDNF), nerve growth factor (NGF), neurotrophin 3, and neurotrophin 4 [20-23]. In particular, BDNF is constitutively expressed in smooth muscle cells and epithelium of the lower respiratory tract; it is taken from the general circulation and stored in human platelets and is released during inflammatory conditions and serum preparation [24]. It’s established that BDNF acts on bronchial hyperresponsiveness by causing neuronal hypersensitivity, thus decreasing the diameter of the airways by enhancement of the basal tone [25]. It was proven to play a contribution in airway obstruction and hyperresponsiveness in a model of allergic asthma [26, 27], and elevated BDNF levels in the serum of patients with COPD were found at all stages, further indicating a role of this molecule in the pathogenesis of diverse pulmonary diseases [24].

The relevance of neurotrophins in lung hyperactivity was found to be particularly important in children with an acute RSV infection in a study carried out in 2005 that found significantly more elevated levels of BDNF and NGF in the bronchoalveolar lavage (BAL) of mechanically ventilated infants positive to RSV, in comparison to mechanically ventilated infants with respiratory failure caused by adenoviral or parainfluenza infections [28, 29]. However, no significant relation between serum BDNF levels and disease severity in RSV-positive infants has been discovered yet.

Thus, the aim and novelty of the present study were to evaluate the serum BDNF, tropomyosin receptor kinase B (TrkB, the main BDNF receptor) and neurofilament light chain protein (Nfl, a biomarker of neurodegeneration) in infants hospitalized with bronchiolitis at discharge and their correlation with the clinical manifestations and RSV infection severity. Indeed, Nfl is a subunit of neurofilaments, which are cylindrical proteins exclusively located in the neuronal axons that can be measured in blood as a marker of neuronal injury [30, 31]. We predicted that BDNF may help in the prediction of the clinical course of bronchiolitis.

## 2 MATERIALS AND METHODS

The patients recruited in our study included 58 infants below 1 year of age admitted to the Pediatric Emergency Room (ER) of the Department of Maternal-Childhood and Urological Sciences of the “Sapienza” University of Rome, Italy, with the diagnosis of bronchiolitis. In the present observational prospective cohort study, the clinical course of bronchiolitis and serum markers’ levels at discharge were studied. As exclusion criteria, we did not recruit infants undergoing previous specific drug treatment such as chemotherapies, anti-inflammatory and immunosuppressants or receiving antibiotic/antifungal therapy up to 15 days prior to enrollment, and, finally, infants suffering from severe other infectious diseases (including SARS-CoV-2), other ongoing inflammatory, cardiovascular, endocrine and autoimmune disorders. Furthermore, we excluded those infants without informed consent by the parents.

The clinical diagnosis was performed based on the presence of respiratory distress, increased work of breathing as shown by intercostal, subdiaphragmatic and jugular retractions, finding of expiratory wheezes and diffuse bilateral crackles on chest auscultation, and the reduced food intake, which was also the main criterion in the decision of hospitalization of these patients.

During the hospitalization, the patients were stratified into 2 groups, defined as mild and severe, according to the Clinical Respiratory Score (CRS) that comprises several predictors of respiratory distress, for example, child’s color, skin, respiratory rate, presence of rales, use of accessory muscles, mental status and oxygen saturation [32, 33]. Supplemental O_2_ was administered if O_2_ saturation levels were persistently below 92% in room air, and the O_2_ supplementation was evaluated as the measure of severity.

All parents were given a questionnaire to collect information about gestational, birth and remote anamnesis, acknowledged that the data obtained would be stored and used in compliance with the laws and regulations in force, and informed consent was obtained. All clinical investigations were conducted according to the Declaration of Helsinki principles. This study population belongs to an Italian cohort of term, healthy infants hospitalized with bronchiolitis at the Department of Maternal Infantile and Urological Sciences, “Sapienza” University of Rome (BROME-Bronchiolitis in Rome-cohort) [34]. The study was approved by the Ethics Committee of the “Policlinico Umberto I” Hospital (No. 2377/2012).

Blood withdrawal was carried out at the moment of infant discharge for both routine analyses, including C-Reactive Protein (CRP) and BDNF/TrkB/Nfl.

### 2.1 Nasopharyngeal Aspirate

A nasopharyngeal aspirate was performed at the entrance to the ward, injecting 1 ml of 0.9% physiological solution into each nostril. The samples were transported on ice within 2 hours to the virology laboratory of the Department of Molecular Medicine of the Sapienza University of Rome. Here, once the mucus was centrifuged and dissolved, the sample was used for isolation and amplification of viral nucleic acid by Reverse Transcriptase - Polymerase Chain Reaction (RT-PCR) or nested PCR methods. Fourteen respiratory viruses were searched (RSV, Influenza A/B, Coronavirus, OC43,229E, NL-63, HUK1, Adenovirus, Rhinovirus, Parainfluenza 1-3, Metapneumovirus and Bocavirus).

### 2.2 BDNF, Nfl and TrkB Serum Analyses

In the pediatric ward, blood samples of 2.5 mL were taken from 58 infants (45%), collected in BD Vacutainer™ Serum Separation Tubes and centrifuged at 3000 rpm for 15 min to separate serum. The serum was then stored at −80°C. BDNF was measured using a sandwich enzyme-linked immunosorbent assay (ELISA) kit (Cat. No. DY248, R&D Systems, Minneapolis, MN, USA), according to the protocols provided by the manufacturer. Serum samples were diluted 100-fold and tested in duplicate. The colorimetric reaction product was measured at 450 nm using a microplate reader (Dynatech MR 5000, PBI International, USA). Data were represented in ng/ml.

TrkB was measured using a sandwich enzyme-linked immunosorbent assay (ELISA) kit (Cat. No. MBS9346917, MyBioSource, Italy) according to the protocols provided by the manufacturer. Serum samples were diluted 2-fold and tested in duplicate. The colorimetric reaction product was measured at 450 nm using a microplate reader (Dynatech MR 5000, PBI International, USA). Data were represented in ng/ml.

Nfl was measured using a sandwich enzyme-linked immunosorbent assay (ELISA) kit (Cat. No. 20-8002, UmanDiagnostics, Sweden) according to the protocols provided by the manufacturer. Serum samples were diluted 4-fold and tested in duplicate. The colorimetric reaction product was measured at 450 nm using a microplate reader (Dynatech MR 5000, PBI International, USA). Data were represented in pg/ml.

### 2.3 Statistical Analysis

Statistical tests were performed with SPSS software (IBM SPSS 18, statistical software). The distribution of the variables was validated by the normality test, and the differences between groups were evaluated using Kruskal-Wallis H to correct for multiple comparisons. The mean for the groups was compared using the one-way ANOVA test or *t*-test as appropriate. Mann-Whitney analyses were used to compare differences between groups in the box plot of the figures. Categorical variables were evaluated by the chi-square test. The correlation between clinical and biochemical data was assessed by Spearman rho correlation analysis. A multiple regression analysis was also used to study the relationship between correlated BDNF and covariates. All the tests were performed with a *p* < .05 as the significance level.

## 3 RESULTS

### 3.1 Patients’ Demographic Characteristics

Table **1** shows the main demographic, clinical and laboratory characteristics of study participants. A total of 58 infants with the diagnosis of bronchiolitis were enrolled in our study, of which 33 (56.9%) were males and 25 (43.1%) were females with an average age of 3.4 months (±2.3 SD), average weight of 5.9 kg (±1.5 SD), and average length 60.8 cm (±5.6 SD). Familial history regarding the presence of atopy was collected from both parents and siblings of each patient, and a positive history was seen in 33 (56.9%) of them. Furthermore, a positive family history of smoking habits was seen in 21.1% of cases. Most of the infants (87.9%) had, in their families, a schooled sibling. RSV-positive infants were 58.6%. No other differences between male and female infants were recorded in the analyzed parameters, and this main factor was pulled out from the statistical outcomes.

### 3.2 BDNF, TrkB and Nfl Investigation

Table **2** displays the multiple regression analysis of BDNF with the main analyzed demographic, clinical and laboratory characteristics (sex, age, smoking parent habits, fever, cough, O_2_, PLT, CRP, RSV). Data disclosed only a significant correlation between BDNF and O_2_ supplementation (*p* < .02). However, no other significant data were evidenced for both TrkB and Nfl (multiple regression analysis data not shown).

**Fig. (1):**
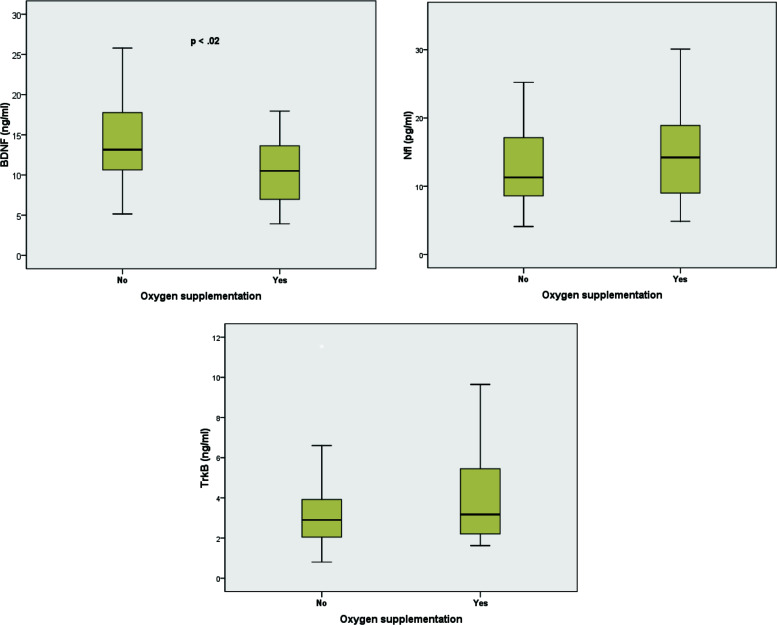
Box plot of serum BDNF (left), Nfl (middle) and TrkB (right) according to Oxygen supplementation. Serum BDNF levels show a significant difference between the two groups (*p* < 0.02; using Mann-Whitney Test). Serum Nfl and TrkB show no significant differences between the two groups. The line in the middle of the boxes represents the median. The horizontal line at the top of the box represents the 75^th^ percentile value. The horizontal line at the bottom of the box represents the 25^th^ percentile value. The line at the top of the whisker represents the upper adjacent value. The line at the bottom of the whisker represents the lower adjacent value. Outlier values are not shown. In particular, the above-mentioned association was found to be stronger in the presence of a positive family history of smoking, fever and diffuse breath sounds.

In particular, a quite interesting statistically significant correlation between oxygen supplementation and decreased BDNF was found in those infants needing O_2_ during hospitalization. Indeed, we set a threshold of BDNF level ≤ 9.0 ng/ml (corresponding to the 1st quartile of the distribution), and out of 19 infants who were treated with O_2_ supplementation, 57.9% had a BDNF below our threshold. Thus, infants with a BDNF value below our threshold had an increased risk of O_2_ supplementation (OR: 3.76, *p* < 0.02, Fig. **1**).

The BDNF serum levels were analyzed in relation to other clinical parameters registered during hospitalization, namely cough, coryza, fever, respiratory distress accompanied by intercostal, jugular and subdiaphragmatic retractions, nasal flaring, wheezing and/or crackles upon physical examination, vomiting, diarrhea and abdominal pain. A remarkable association, but without full significance, was found between BDNF and the clinical outcomes of the disease during the hospitalization, as a matter of clinical presentation demonstrated intercostal, subdiaphragmatic and jugular retractions, presence of fever, and localized and/or diffuse wheezing upon auscultation. In particular, when we set the threshold of BDNF level < 9.0 ng/ml, a statistically significant correlation was found between infants with BDNF below the threshold and the presence of fever during hospitalization (Table **3**).

As shown in Fig. (**2**), Spearman correlations between BDNF and the blood routine analyses clearly also show also a positive association with platelets (PLT) and a negative with CRP, ps < .05.

## 4 DISCUSSION

To the best of our knowledge, this is the first study to demonstrate that infants with severe bronchiolitis show at hospital discharge low levels of BDNF in the serum. No changes were observed in both TrkB (the main BDNF receptor) and Nfl (a biomarker of neurodegeneration). In addition to its notable role in the nervous system, BDNF, as well as their receptors [35-38], are expressed in the lung crucially contributing to both normal physiology and pathophysiology of several pulmonary diseases [39]. The relevance of BDNF lies in novel clinical findings indicating changes in this neurotrophin and function in a variety of disorders including neonatal and adult asthma, influenza (including COVID-19), sinusitis, and lung tumor [39-43]. In particular, it has shown the significance of BDNF expression and signaling mechanisms in lung development and early airway, crucial in neonatal lung function and also its change in prematurity and insults such as infection and inflammation [39]. Data also display that BDNF originating from airway nerves subtly regulates neurogenic control of airway abilities, also during allergic inflammation-induced dysfunctional outcomes [44].

Despite the large number of studies regarding this issue, bronchiolitis’ clinical course remains very unpredictable and vastly variable, ranging from a mildly symptomatic disease, manageable at home, to a very severe pathology that may require O_2_ supplementation and sometimes admission in the pediatric intensive care unit [45-47].

Given the lack of clear and defined prognostic factors, our study aimed at finding and analyzing relevant indexes that could potentially help in predicting a more or less severe clinical course during the hospitalization by analyzing the clinical progression in infants who were admitted to the hospital with the diagnosis of bronchiolitis. In our study, a relevant role was found in the lower serum BDNF level at the time of discharge. Indeed, a remarkable association was demonstrated in infants who needed oxygen supplementation during hospitalization: indeed, a significant number of them, approximately 80%, was found to have a BDNF level below our cutoff value.

The exact mechanism underlying a decreased BDNF level in the serum of these patients remains unclear. However, speculations meant to explain the low BDNF in infants with severe bronchiolitis may be proposed. As a first hypothesis, the serum BDNF reduction could be associated with a higher utilization by lung cells, but this assumption does not correlate with the presence of serum TrkB. As a second hypothesis, the decrease in BDNF could be due to an altered production by lung cells. Therefore, further research is required to study if a decreased production or increased consumption of this biomarker is at the base of the above-mentioned findings.

As for BDNF in RSV-induced infection, only a few studies were published [19]. A previous study on respiratory syncytial virus-infected mice showed BDNF elevation in the lung during the period of the infection [48]. However, in a study on eight individuals affected by respiratory bronchiolitis-associated interstitial lung disease, no changes in BDNF were revealed, but a faint expression of neurotrophin-3 and other neurotrophin receptors was detected [23].

The changes in BDNF at the time of discharge might lead to additional pieces of discussion and future research. Indeed, oxidative stress (OS) plays a crucial role in the pathogenesis of inflammatory lung diseases. Infants hospitalized for bronchiolitis are at high risk for asthma. Glutathione-related metabolites may antagonize oxidative stress, which induces airway injuries in respiratory infection and subsequent airway remodeling. However, little is known about the relationship of glutathione-related metabolites with bronchiolitis severity and the risk of asthma. In a multicenter prospective observational cohort study of infants hospitalized for bronchiolitis, nasopharyngeal and serum glutathione-related metabolites were measured by using liquid chromatography-tandem mass spectrometry, with the outcome demonstration that bronchiolitis-hospitalized infants exhibited changes in glutathione-related metabolites being associated with bronchiolitis severity and asthma risk [49]. This points to a possible involvement of ferroptosis *via* the BDNF/Nrf2 axis. Nuclear factor E2-related factor 2 (Nrf2) is a key regulator of antioxidant response and is considered to be an important therapeutic target for oxidant-induced inflammatory and/or degenerative diseases. In recent studies, the selenated derivative of antioxidant polyphenols has been synthesized, showing a significant cryoprotective effect.

Bronchiolitis and the associated degenerative damage is a multifaceted biological process involving various genetic, environmental, and lifestyle factors. The major factor in this process is oxidative stress, caused by an abundance of reactive oxygen species (ROS) generated in the mitochondria and endoplasmic reticulum (ER). ROS and reactive nitrogen species (RNS) pose a threat by disrupting signaling mechanisms and causing oxidative damage to cellular components. This oxidative stress affects both the ER and mitochondria, causing initiation of unfolded protein response, and mitochondrial dysfunction, ultimately leading to inflammaging of the respiratory tract. RONS during oxidative stress dysregulate multiple metabolic pathways like NF-κB, Mitogen-activated protein kinase (MAPK), Nrf-2/Keap-1/ARE and PI3K/Akt, which may lead to apoptosis and/or ferroptosis or necrosis depending on metabolomics conditions.

Consistent with this notion, infants with bronchiolitis are at increased risk for developing asthma, and growing evidence suggests bronchiolitis is a heterogeneous condition [50, 51]. By applying an unsupervised clustering approach to the nasopharyngeal airway metabolome data from a multicenter prospective cohort study of infants with severe bronchiolitis, there have been identified 5 biologically distinct and clinically meaningful metabotypes. Specifically, the metabotype characterized by a high abundance of inflammatory amino acids and a low abundance of polyunsaturated fatty acids (PUFAs) had the highest risk for developing asthma. These important data lend significant support to the concept that bronchiolitis is a heterogenous syndrome with different biological mechanisms. These observations should facilitate further investigations into the development of metal-type-targeted strategies for bronchiolitis treatment and asthma prevention.

Furthermore, future examinations of the relationship between bronchiolitis metabotypes and asthma endotypes (e.g., based on the genome, transcriptome, and metabolome) will also provide a new avenue for the development of endotypes-specific prevention strategies for asthma. Thus, relevant to bronchiolitis pathophysiology and its therapeutics, redox-active compounds, which have been shown to act *via* hormetic dose responses, are conceivable eligible due to their powerful anti-inflammatory effects, displaying endpoints of biomedical and clinical relevance. Thus, the interplay and coordination of redox interactions and their interaction with endogenous and exogenous antioxidant defense systems is an emerging area of research interest in anti-inflammatory anti-degenerative therapeutics [52-54].

We did not detect modifications in Nfl in the enrolled infants. Nfl has a key role in the assembling of neurofilaments, which are only present in neurons where they are the main structural proteins, particularly concentrated in large projection axons [46, 47]. Indeed, axons are particularly sensitive to metabolic and mechanical alterations, and as a result, axonal deterioration is a substantial trouble in many neurological diseases [46, 47]. Thus, the present findings do suggest that in the enrolled infants, luckily, no gross neurobiological disruptions were induced by RSV infection.

This study obviously has some limitations. The *n* of infants was different and small (but this also depends on the quite delimited inclusion/exclusion criteria of the investigation and because the results of this study originate from a single University hospital), so some biases could have arisen. Also, the absence of controls needed for the comparison of BDNF levels in our sample may be considered a limitation in the understanding of the role of BDNF as a biomarker of the clinical course of bronchiolitis and as a prognostic index. However, it should be noted that blood withdrawal in healthy infants is very difficult due to ethical problems (at least in Italy) and because parents do not easily provide permission and informed content for so young kids. This ethical/familial issue was also the reason for the single blood withdrawal for each infant.

## CONCLUSION

In conclusion, this study shows that infants affected by bronchiolitis needing O_2_ supplementation decreased BDNF serum levels at the time of discharge. However, further studies are necessary to confirm and extend the fact that BDNF in the serum could be considered a key biomarker of lung inflammation to unveil another risk factor for severe bronchiolitis. The present study also provides an additional step in the study of RSV-induced bronchiolitis for evaluating other appropriate biomarkers in order to early disclose infants at higher risk. Furthermore, these findings might be of interest to scholars involved in the field of human pulmonary diseases caused by bacterial or viral infections.

## Figures and Tables

**Fig. (2):**
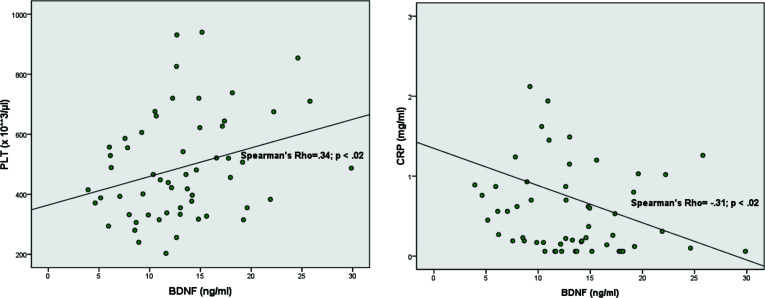
Correlations according to Spearman between serum BDNF and PLT (left) and CRP (right). The Rho and P values are indicated in the box.

**Table 1:** Main demographic, clinical and laboratory characteristics of study participants.

**Variables**	**Males (n = 33)**	**Females (n = 25)**	*p*-Value (*)	**Total (n = 58)**
Age (Months)	3.5 ± 2.2	3.3 ± 2.5	.78	3.4 ± 2.3
Weight (Kg)	6.2 ± 1.5	5.6 ± 1.4	.08	5.9 ± 1.5
Length (cm)	62.3 ± 6.2	58.7 ± 4.0	**.02**	60.8 ± 5.6
Fever (%)	54.5	54.2	.97	54.4
Cough (%)	97.0	91.7	.38	94.7
Intercostal retractions (%)	72.7	87.5	.18	78.9
Subdiaphragmatic retractions (%)	90.9	87.5	.68	89.5
Jugular retractions (%)	60.6	66.7	.64	63.2
Localized wheezing (%)	12.1	20.8	.37	15.8
Diffuse wheezing (%)	66.7	45.8	.12	57.9
Oxygen supplementation (%)	30.3	37.5	.57	33.3
CRS > 1 (%)	69.7	66.7	.80	68.4
Antibiotics (%)	24.2	8.7	.13	17.9
Bronchodilators (%)	75.8	66.7	.45	71.9
BDNF (ng/ml) (#)	12.6 ± 4.7	13.8 ± 6.2	.90	13.1 ± 5.4
Nfl (pg/ml) (#)	12.8 ± 5.8	13.3 ± 6.2	.61	13.0 ± 5.9
TrkB (ng/ml) (#)	3.9 ± 3.4	4.1 ± 2.7	.36	4.0 ± 3.1
PLT (x 10**3/µl)	533 ± 188	427 ± 136	.22	470 ± 163
CRP (mg/ml)	.67 ± 0.9	.83 ± 1.4	.88	.77 ± 1.18
RSV + (%)	58.1	80.0	.10	66.7

**Note:** P positive values between groups are shown in bold. # Data for BDNF, TrkB and Nfl are expressed for n = 58; males = 33; females = 25.

**Abbreviations**: CRS – clinical respiratory scores; BDNF – brain-derived neurotrophic factor; Nfl – neurofilament light chain; TrkB - tropomyosin receptor kinase B; CRP – C-reactive protein; PLT – platelets; RSV - respiratory syncytial virus. (*) Mann-Whitney independent groups test or Chi-Square test.

**Table 2:** Multiple regression analysis of BDNF with the main analyzed demographic, clinical and laboratory characteristics (sex, age, smoking parent habits, fever, cough, O_2_ supplementation, PLT, CRP, RSV).

**Model**		**R**			**R Square**			**Adj. R Square**	
1		.571^a^			.326			.178	
**ANOVA^b^**									
**Model**			**Sum of Squares**	**df**		**Mean Square**	**F**		**Sig.**
1	Regression		478.284	9		53.143	2.200		**.042** ^a^
	Residual		990.412	41		24.156	-		-
	Total		1468.696	50		-	-		-

**Note: **^a^Predictors: (Constant). Males. Age (months). Smoking Parents. Fever. Cough. Oxygen supplementation. PLT (x 10**3/µl). CRP (mg/ml). RSV+, ^b^Dependent Variable: BDNF (ng/ml).

**Table:** 

**Coefficients^a^**								
**Model**		**Unstandardized ** **Coefficients**		**Standardized ** **Coefficients**	**t**	**Sig.**	**95.0% Confidence Interval for B**	
		**B**	**Std. Error**	**Beta**			**Lower Bound**	**Upper Bound**
1	(Constant)	16.213	4.228	-	3.835	.000	7.675	24.750
	Males	-1.876	1.432	-.173	-1.310	.197	-4.768	1.016
	Age (months)	.205	.383	.077	.535	.595	-.569	.979
	Smoking parents	-2.361	1.634	-.189	-1.445	.156	-5.661	.939
	Fever	-2.039	1.653	-.188	-1.233	.224	-5.377	1.300
	Cough	-3.957	3.123	-.168	-1.267	.212	-10.265	2.350
	Oxygen supplementation	-3.565	1.486	-.319	-2.399	**.021**	-6.567	-.564
	PLT (x 10**3/µl)	.008	.004	.242	1.824	.075	-.001	.017
	CRP (mg/ml)	-.483	.638	-.105	-.756	.454	-1.771	.806
	RSV +	1.010	1.516	.090	.666	.509	-2.051	4.071

**Note: **^a^Dependent Variable: BDNF (ng/ml).

**Abbreviations:** BDNF – brain-derived neurotrophic factor; CRP – C-reactive protein; PLT – platelets; RSV - respiratory syncytial virus.

**Table 3:** Relationship between BDNF and the infant clinical manifestations and therapy.

**-**	**BDNF ≤ 9.0 ng/ml**	**BDNF > 9.0 ng/ml**	***p*-value**
Fever	78.6%	46.5%	**.036**
Intercostal retractions	92.9%	74.4%	.14
Subdiaphragmatic retractions	92.9%	88.4%	.635
Jugular retractions	78.6%	58.1%	.169
Localized wheezing	21.4%	14%	.505
Diffuse wheezing	71.4%	53.5%	.238
Clinical Respiratory Score (CRS) >1	85.7%	62.8%	.109
Oxygen supplementation	57.1%	26.2%	**.034**
Antibiotics	7.1%	21.4%	.23
Bronchodilators	85.7%	67.4%	.19

**Note: ***P* positive values are shown in bold.

## Data Availability

The data that support the findings of this study are available from the corresponding authors, (MF, FM), upon reasonable request.

## LIST OF ABBREVIATIONS

BDNF: Brain-derived Neurotrophic Factor
ELISA: Enzyme-linked Immunosorbent Assay
ER: Endoplasmic Reticulum
NGF: Nerve Growth Factor
Nfl: Neurofilament Light Chain
OS: Oxidative Stress
PUFAs: Polyunsaturated Fatty Acids
RNS: Reactive Nitrogen Species
ROS: Reactive Oxygen Species
RSV: Respiratory Syncytial Virus
